# RANKL inhibition by osteoprotegerin prevents bone loss without affecting local or systemic inflammation parameters in two rat arthritis models: comparison with anti-TNFα or anti-IL-1 therapies

**DOI:** 10.1186/ar2879

**Published:** 2009-12-11

**Authors:** Marina Stolina, Georg Schett, Denise Dwyer, Steven Vonderfecht, Scot Middleton, Diane Duryea, Efrain Pacheco, Gwyneth Van, Brad Bolon, Ulrich Feige, Debra Zack, Paul Kostenuik

**Affiliations:** 1 Department of Metabolic Disorders, Amgen Inc., One Amgen Center Drive, Thousand Oaks, CA 91320, USA; 2 Department of Inflammation, Amgen Inc., One Amgen Center Drive, Thousand Oaks, CA 91320, USA; 3 Current address: Department of Internal Medicine 3, University of Erlangen-Nuremberg, Glückstrasse 4a, 91054 Erlangen, Germany; 4 Department of Pathology, Amgen Inc., One Amgen Center Drive, Thousand Oaks, CA 91320, USA; 5 Current address: GEMpath, 2867 Humboldt Circle, Longmont, CO 80503, USA; 6 Current address: EUROCBI GmbH, Bodenacherstrasse 87, 8121 Benglen-Zurich, Switzerland

## Abstract

**Introduction:**

Rat adjuvant-induced arthritis (AIA) and collagen-induced arthritis (CIA) feature bone loss and systemic increases in TNFα, IL-1β, and receptor activator of NF-κB ligand (RANKL). Anti-IL-1 or anti-TNFα therapies consistently reduce inflammation in these models, but systemic bone loss often persists. RANKL inhibition consistently prevents bone loss in both models without reducing joint inflammation. Effects of these therapies on systemic markers of bone turnover and inflammation have not been directly compared.

**Methods:**

Lewis rats with established AIA or CIA were treated for 10 days (from day 4 post onset) with either PBS (Veh), TNFα inhibitor (pegsunercept), IL-1 inhibitor (anakinra), or RANKL inhibitor (osteoprotegerin (OPG)-Fc). Local inflammation was evaluated by monitoring hind paw swelling. Bone mineral density (BMD) of paws and lumbar vertebrae was assessed by dual X-ray absorptiometry. Markers and mediators of bone resorption (RANKL, tartrate-resistant acid phosphatase 5b (TRACP 5B)) and inflammation (prostaglandin E_2 _(PGE_2_), acute-phase protein alpha-1-acid glycoprotein (α_1_AGP), multiple cytokines) were measured in serum (day 14 post onset).

**Results:**

Arthritis progression significantly increased paw swelling and ankle and vertebral BMD loss. Anti-TNFα reduced paw swelling in both models, and reduced ankle BMD loss in AIA rats. Anti-IL-1 decreased paw swelling in CIA rats, and reduced ankle BMD loss in both models. Anti-TNFα and anti-IL-1 failed to prevent vertebral BMD loss in either model. OPG-Fc reduced BMD loss in ankles and vertebrae in both models, but had no effect on paw swelling. Serum RANKL was elevated in AIA-Veh and CIA-Veh rats. While antiTNFα and anti-IL-1 partially normalized serum RANKL without any changes in serum TRACP 5B, OPG-Fc treatment reduced serum TRACP 5B by over 90% in both CIA and AIA rats. CIA-Veh and AIA-Veh rats had increased serum α_1_AGP, IL-1β, IL-8 and chemokine (C-C motif) ligand 2 (CCL2), and AIA-Veh rats also had significantly greater serum PGE_2_, TNFα and IL-17. Anti-TNFα reduced systemic α_1_AGP, CCL2 and PGE_2 _in AIA rats, while anti-IL-1 decreased systemic α_1_AGP, IL-8 and PGE_2_. In contrast, RANKL inhibition by OPG-Fc did not lessen systemic cytokine levels in either model.

**Conclusions:**

Anti-TNFα or anti-IL-1 therapy inhibited parameters of local and systemic inflammation, and partially reduced local but not systemic bone loss in AIA and CIA rats. RANKL inhibition prevented local and systemic bone loss without significantly inhibiting local or systemic inflammatory parameters.

## Introduction

Rheumatoid arthritis (RA) is an immune-mediated disease that affects synovial membranes, articular cartilage, and bone. Arthritis progression is associated with chronic soft tissue inflammation, which is commonly followed by joint destruction. RA is initiated and maintained by interacting cascades of proinflammatory cytokines [1,2]. TNFα and IL-1 are key mediators of inflammation in patients with inflammatory arthritis [3-6]. Their central importance is demonstrated by the ability of anti-TNFα and anti-IL-1 therapies to markedly reduce clinical and structural measures of disease in arthritic patients [7,8] and in animals with induced arthritis [9-14]. While inhibition of IL-1 or TNFα yields significant anti-inflammatory effects in rats with adjuvant-induced arthritis (AIA) [10,15,16] and in human arthritis [17-19], focal bone erosions in affected joints and systemic bone loss are not fully prevented.

Focal bone erosions within inflamed joints are a hallmark of immune-mediated arthritis and have been attributed to excessive osteoclast activity [20-22] mediated primarily by receptor activator of NF-κB ligand (RANKL), also known as osteoclast differentiation factor (ODF), osteoprotegerin (OPG) ligand (OPGL), and TNF-related activation-induced cytokine (TRANCE). RANKL is an essential mediator of bone resorption. RANKL and its natural inhibitor OPG play important roles in the skeletal deterioration associated with RA [23]. In animal models, RANKL inhibition with recombinant OPG inhibits bone erosions in rats with AIA or collagen-induced arthritis (CIA) [16,21,24-26], and in transgenic mice overexpressing TNFα [27,28]. TNFα and IL-1β have been shown to stimulate RANKL expression [29,30], which could contribute to the increases in RANKL and to the bone erosions that have been documented in rats with CIA or AIA [31] and in arthritic patients [32]. Consistent with this, anti-TNFα therapy has been shown to significantly reduce serum RANKL in arthritic patients [32]. The effects of anti-IL-1 therapy on serum RANKL have not been previously examined in arthritis settings, and were therefore a focus of the current study.

In addition to focal bone erosions, inflammatory arthritis is also a systemic disease characterized by bone loss in locations away from affected joints [28,33-35], increased serum concentrations of bone turnover markers [36], and increased concentrations of circulating markers and mediators of inflammation [36-39]. To date, there are only limited data regarding the effects of anti-TNFα, anti-IL-1 or anti-RANKL therapies on systemic bone loss in arthritis patients [40], and there are no comparative data on the effects of these therapies on systemic markers or mediators of inflammation in either human or preclinical models.

Arthritis progression in two rat models - AIA and CIA - is thought to arise from distinct immunopathogenic mechanisms [41], a notion recently substantiated by descriptions of their divergent cytokine profiles [38,39]. The current studies were therefore conducted in rats with AIA or CIA to compare and contrast the effects of specifically inhibiting TNFα, IL-1 or RANKL on local and systemic bone loss, and on systemic markers and mediators of inflammation. The novelty of the current study is based on the fact that these therapies were introduced at the peak of the clinical phase of arthritis to more closely model the clinical scenarios where they might be administered to human patients, in contrast to previous publications where treatments were already started at the onset of arthritis. We hypothesized that RANKL inhibition would prevent local and systemic bone loss without inhibiting systemic markers and mediators of inflammation in both AIA and CIA rats, while inhibition of IL-1 or TNFα would suppress systemic levels of proinflammatory cytokines in these two arthritis models. Furthermore, based on the ability of TNFα and IL-1 to directly induce RANKL expression, we hypothesized that inhibition of TNFα or IL-1 would indirectly act to reduce RANKL levels in arthritic rats.

## Materials and methods

### Animals

Lewis rats (7 to 8 weeks old; Charles River Laboratories, Wilmington, MA, USA) were acclimated for 1 week and then randomly assigned to treatments (see below). Animals received tap water and pelleted chow (#8640, Harlan Laboratories, Indianapolis, IN, USA) *ad libitum*; the calcium and phosphorus contents were 1.2% and 1.0%, respectively. These studies were conducted in accordance with federal animal care guidelines and were pre-approved by the Institutional Animal Care and Use Committee of Amgen Inc.

### Induction of arthritis

Both AIA and CIA were induced as detailed previously [10,16]. Briefly, AIA was incited in male rats by a single intradermal injection into the tail base of 0.5 mg heat-killed mycobacteria H37Ra (Difco, Detroit, MI, USA) suspended in paraffin oil. CIA was elicited in female rats by intradermal injections (at 10 sites scattered over the back) of porcine type II collagen (1 mg total; Chondrex, Redmond, WA, USA) emulsified 1:1 with Freund's incomplete adjuvant (Difco).

### Treatments

For both the CIA and AIA models, rats were randomly assigned to one of the following single-agent treatment groups (n = 8/group): PEGylated soluble TNF receptor type I (pegsunersept) at 4 mg/kg/day (by daily subcutaneous bolus), IL-1 receptor antagonist (anakinra) at 100 mg/kg/day (by subcutaneous infusion using an Alzet osmotic minipump; Durect Corp., Cupertino, CA, USA), or a modified version of OPG (consisting of the RANKL-binding portion of OPG linked with the constant (Fc) domain of IgG) at 3 mg/kg/day (given every other day by subcutaneous bolus). All molecules were fully human recombinant proteins made by Amgen Inc. (Thousand Oaks, CA, USA). In addition, each model included a vehicle (Veh) control group (PBS, pH 7.4, given by daily subcutaneous bolus). Doses for all agents were selected based on the levels established in previous studies [10,15]. Treatments were started 4 days after the onset of clinical disease (that is, after both local inflammation and erosion were well established) [16,38,39] and were continued for 10 days.

### Evaluation of paw swelling as a parameter of arthritis-induced local inflammation

Hind paw swelling was examined by measuring the average hind paw volume via water plethysmography (for AIA rats) [10] or measuring the hind paw diameter via precision calipers (for CIA rats) [31].

### Histology and immunohistochemistry of ankles and vertebrae

At the end of the study (day +14 post onset), the left ankle and lumbar vertebrae were removed, fixed by immersion in zinc formalin, decalcified in eight serial changes of a 1:4 mixture of 8 N formic acid and 1 N sodium formate for approximately 1 week, trimmed along the longitudinal axis, and processed into paraffin. Sections (3 μm) were deparaffinized, pretreated with Antigen Retrieval Citra (BioGenex, San Ramon, CA, USA), incubated with polyclonal anti-cathepsin K antibody (Amgen Inc.) at 1 μg/ml for 1 hour at room temperature, and then quenched with 3% H_2_O_2_. The location of the anti-cathepsin K antibody was detected by EnVision Labelled Polymer Horseradish Peroxidase (Dako, Carpenteria, CA, USA) followed by application of diaminobenzidine (Dako). All sections were counterstained with H & E for analyses.

Histology slides were examined by routine light microscopy, and the severity of inflammatory cell infiltration was scored using a tiered, semi-quantitative scale: 0 = no infiltrate; 1 = minimal (few cells in perisynovial and synovial tissues); 2 = mild (infiltrating cells more numerous in perisynovial and synovial tissues, and/or in bone marrow immediately beneath joints; occasional small clusters of inflammatory cells); 3 = moderate (inflammatory cell infiltrate more intense in perisynovial and synovial tissues, and often extending into adjacent periosseous tissues including ligaments, tendons, and skeletal muscle and/or in bone marrow immediately beneath joints; occasional dense aggregates of inflammatory cells); and 4 = marked (increasing intensity of inflammatory cell infiltrate in synovial and perisynovial tissues, and extending into adjacent periosseous tissues and/or widely dispersed in bone marrow; often several dense aggregates of inflammatory cells). The slides were examined without knowledge of the treatment group on two occasions separated by several days.

### Bone mineral density evaluation

Left ankle areal bone mineral density (BMD) and vertebral BMD were measured in anesthetized rats on the day of arthritis onset (day 0) and at the end of the study (day 14) by dual X-ray absorptiometry (QDR 4500a; Hologic, Inc., Bedford, MA, USA).

### Biochemical evaluation of serum markers and mediators

Separate aliquots of terminal serum were used to quantify levels of various analytes. The serum concentration of OPG-Fc was assessed individually for OPG-Fc-treated animals by an inhouse-developed ELISA (Amgen Inc.). Briefly, ELISA plates were precoated with mouse anti-human IgG (Abcam, Cambridge, MA, USA) as a capture reagent, incubated overnight at 4°C and blocked for 1 hour at room temperature with a 1% BSA solution in PBS (Kirkegaard and Perry Laboratories Inc., Gaithersburg, MD, USA). Standards (human OPG-Fc generated inhouse; Amgen Inc.) and study samples were loaded into the wells and incubated for 1 hour at room temperature. After a wash step, horseradish peroxidase-conjugated anti-human OPG detection antibody (generated inhouse; Amgen Inc.) was added and incubated at room temperature for 1 hour. Following a final wash step, a tetramethylbenzidine-peroxidase substrate (Kirkegaard and Perry Laboratories Inc.) was added to the plate. The reaction, visualized by color development, was stopped with 2 M sulfuric acid and the absorbance (optical density) was measured at 450 nm wavelength (SpectraMax M5 plate reader; Molecular Devices Corp., Sunnyvale, CA, USA). The conversion of optical density units for the study samples to concentration was achieved through a computer software-mediated comparison with a standard curve developed during the same analytical run using four-parameter curve-fitting software (Softmax Pro; Molecular Devices Corp.).

The major rat acute-phase protein alpha-1-acid glycoprotein (α_1_AGP) was measured with a precipitin ring assay (Ecos Institute, Miyagi, Japan). Prostaglandin E_2 _(PGE_2_) was evaluated using an enzyme immunoassay kit (Cayman Chemical, Ann Arbor, MI, USA).

Multiple cytokines (RANKL, OPG, chemokine C-C motif ligand 2 (CCL2), IL-17, TNFα, IL-8 and IL-1β) and C-reactive protein were assessed using multiplex or singleplex, rat-specific Luminex kits (Linco Research, St Charles, MO, USA). Due to the interference of pharmacological concentrations of OPG-Fc with capture and/or detection antibodies used for RANKL and OPG assays, we were not able to effectively evaluate serum levels of rat RANKL and rat OPG in samples collected from OPG-Fc-treated animals.

The serum concentration of the bone resorption marker tartrate-resistant acid phosphatase 5b (TRACP 5B) was evaluated by enzyme immunoassay (RatTRAP; SBA Sciences, Oulu, Finland).

All of the commercial assays were performed according to the manufacturers' instructions.

### Regression analyses

Correlations of the BMD percentage change or the paw swelling percentage change versus serum concentrations of RANKL, TNFα or IL-1β were established using linear regression analysis (GraphPad Prism software, GraphPad Software, Inc., La Jolla, CA, USA).

### Statistical analyses

Data represent means ± standard error of the means. Comparisons between the groups were made by one-way analysis of variance followed by Dunnett's post-test, with *P *< 0.05 indicating statistical significance. Comparisons were made for each group versus nonarthritic controls, versus arthritic vehicle-treated animals, or versus arthritic OPG-Fc-treated animals, as indicated.

## Results

### Effects of anti-TNFα, anti-IL-1, or anti-RANKL therapy on local joint inflammation

**Figure 1:**
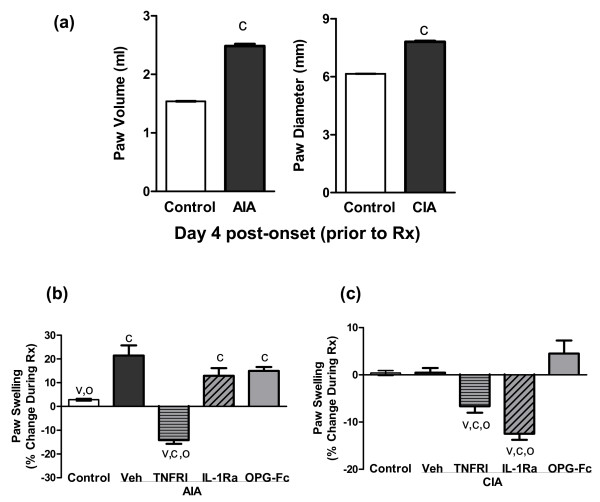
Effect of anti-TNFα, anti-IL-1, or anti-RANKL therapy on hind paw swelling. Effect of PEGylated soluble TNF receptor type I (TNFRI), IL-1 receptor antagonist (IL-1Ra) or osteoprotegerin (OPG)-Fc on hind paw swelling. **(a) **Hind paw swelling was assessed on day 4 post onset, prior to the beginning of therapies. Swelling was assessed in adjuvant-induced arthritis (AIA) rats by measuring average hind paw volume via water plethysmography, and in collagen-induced arthritis (CIA) rats by precision caliper measurements of paw diameter. **(b), (c) **Percentage changes in paw swelling in AIA and CIA rats, as measured from the time of treatment initiation (day 4) to day 14. Data represent means ± standard error of the means, n = 8/group. ^c^Significantly different from control (nonarthritic) rats, *P *< 0.05. ^v^Significantly different from vehicle (Veh)-treated arthritic rats, *P *< 0.05. ^o^Significantly different from osteoprotegerin-treated arthritic rats, *P *< 0.05.

Based on previously reported results [16,38,39], anti-TNFα, anti-IL-1, or anti-RANKL therapy was begun on day 4 after initial onset of arthritis, when paw swelling was at or near its peak in both CIA and AIA rats (Figure 1a). At this time point, the paw volume was increased by 70% in AIA rats (compared with non-AIA controls, *P *< 0.05) while the paw diameter was increased by 30% in CIA animals (*P *< 0.05 compared with non-CIA controls). After 10 days of treatment with anti-TNFα, paw swelling was significantly reduced in AIA rats, an effect that ultimately reversed much of the swelling that developed prior to treatment (Figure 1b). In contrast, there was no significant effect of anti-IL-1 or anti-RANKL therapy on paw swelling in AIA rats. In CIA rats, anti-IL-1 therapy induced significant reversal of paw swelling, which resulted in near normalization of paw dimensions (Figure 1c). Anti-TNFα therapy partially corrected paw swelling in CIA rats, although less effectively than anti-IL-1. Anti-RANKL therapy had no significant effect on paw swelling in CIA rats.

**Figure 2:**
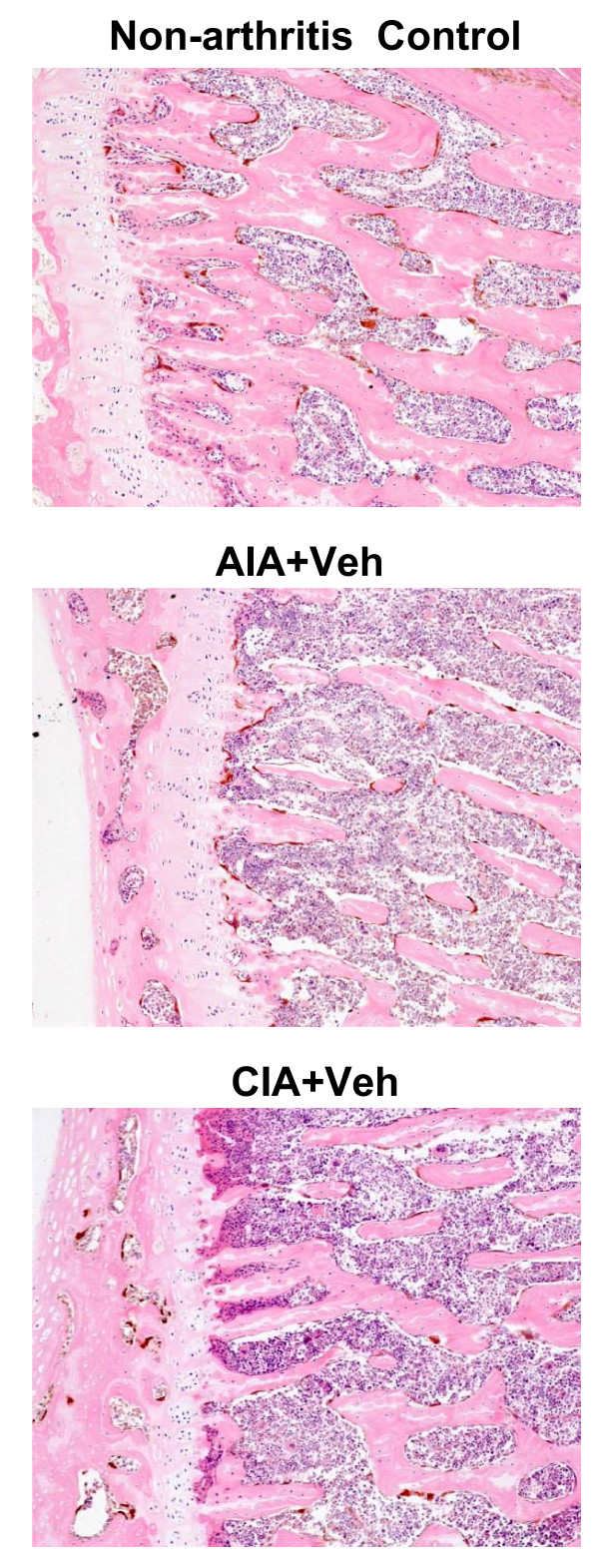
Lumbar vertebrae from nonarthritic control and vehicle-treated adjuvant-induced arthritis and collagen-induced arthritis rats. Representative photomicrographs of lumbar vertebrae from nonarthritic control and vehicle (Veh) (PBS)-treated adjuvant-induced arthritis (AIA) and collagen-induced arthritis (CIA) rats. The trabeculae beneath the physeal plate (pale vertical column at the left margin) were attenuated in arthritic animals but the bone marrow composition and density - including the population of subphyseal osteoclasts (brown cells, cathepsin K-positive) - were equivalent among nonarthritic and arthritic animals. Stain: immunohistochemistry for cathepsin K with H & E counterstain. Magnification: ×100.

The results of histological evaluation of rat ankles for inflammation are summarized in Table 1. Inflammatory cell infiltration into and around affected joints was decreased in CIA rats treated with anti-TNFα or anti-IL-1. A similar effect on inflammatory cell infiltration was not seen with anti-RANKL treatment of CIA rats. None of the therapies reduced the extent of inflammatory cell infiltration into arthritic joints of AIA rats. In contrast to the ankles, inflammation was absent in histological sections from vertebra in AIA and CIA groups treated with vehicle (Figure 2) or in sections from anti-TNFα-treated, anti-IL-1-treated and anti-RANKL-treated groups (data not shown). The latter finding indicated that inflammation was not a prominent feature of skeletal pathology at sites far distant from arthritic joints.

**Table 1:** Histological evaluation of inflammation in rat hind paws

Treatment group	Inflammation score	
	
	Adjuvant-induced arthritis	Collagen-induced arthritis
Nonarthritis control	0^†,‡^	0^†,‡^
Arthritis + vehicle	3.8 ± 0.1*	3.1 ± 0.1*
Arthritis + PEGylated soluble TNF receptor type I	3.6 ± 0.3*	2.0 ± 0.3* ^,†,‡^
Arthritis + IL-1 receptor antagonist	3.8 ± 0.2*	1.2 ± 0.1* ^,†,‡^
Arthritis + osteoprotegerin-Fc	3.8 ± 0.2*	3.4 ± 0.2*

Data represent mean ± standard error of the means, n = 8/group. *Significantly different from control (nonarthritic) rats, *P *< 0.05. ^†^Significantly different from vehicle-treated arthritic rats, *P *< 0.05. ^‡^Significantly different from osteoprotegerin-treated arthritic rats, *P *< 0.05.

### Effects of anti-TNFα, anti-IL-1, or anti-RANKL therapy on local and systemic bone loss

**Figure 3:**
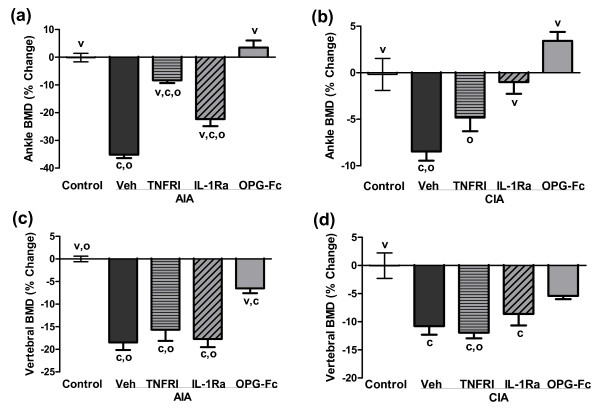
Effect of anti-TNFα, anti-IL-1, or anti-RANKL therapy on bone mineral density. Effects of PEGylated soluble TNF receptor type I (TNFRI), IL-1 receptor antagonist (IL-1Ra) or osteoprotegerin (OPG)-Fc on areal bone mineral density (BMD) of the **(a), (b) **ankle and **(c), (d) **lumbar vertebrae. Baseline BMD measures were obtained by dual X-ray absorptiometry on the day of onset for clinical arthritis (day 0). Treatments were initiated on day 4, and final BMD was measured on day 14 post onset. Data represent means ± standard error of the means, n = 8/group. ^c^Significantly different from control (nonarthritic) rats, *P *< 0.05. ^v^Significantly different from vehicle (Veh)-treated arthritic rats, *P *< 0.05. ^o^Significantly different from OPG-treated arthritic rats, *P *< 0.05. AIA, adjuvant-induced arthritis; CIA, collagen-induced arthritis.

Local bone loss within inflamed hind paws was evaluated by dual X-ray absorptiometry analysis, with data presented as the percentage change in ankle BMD from day 0 (onset of arthritis) to the end of the study (day 14 post onset). Ankle BMD was reduced by 35% in vehicle-treated AIA rats, and by 8.5% in vehicle-treated CIA rats (Figure 3a, b; both *P *< 0.05 versus healthy controls).

Treatment of AIA rats with anti-TNFα or anti-IL-1 reduced ankle BMD loss, although these treatments did not provide full protection as compared with healthy controls (Figure 3a). The preservation was more modest for anti-IL-1. In contrast, anti-RANKL therapy fully prevented ankle BMD loss (Figure 3a). In the CIA rat model, anti-TNFα therapy yielded modest prevention of ankle BMD loss (nonsignificant versus healthy controls; Figure 3b), anti-IL-1 therapy provided nearly complete protection of ankle BMD, and anti-RANKL therapy fully prevented ankle BMD loss (Figure 3b).

As with the ankle, vertebral BMD loss was greater in the AIA model compared with the CIA model (-18.5% vs. -10.8%, respectively, relative to healthy controls). Unlike the ankle, however, neither anti-TNFα nor anti-IL-1 therapies significantly prevented vertebral BMD loss in either model (Figure 3c, d). In contrast, anti-RANKL therapy afforded partial but significant preservation of vertebral BMD in AIA rats (Figure 3c), and nonsignificant preservation of vertebral BMD in CIA rats (Figure 3d). It is noteworthy that initial dual X-ray absorptiometry measurements were obtained 4 days prior to the initiation of treatments, by which time some irreversible bone loss might have already occurred.

### Effects of anti-TNFα, anti-IL-1, or anti-RANKL therapy on serum RANKL and TRACP 5B

**Figure 4:**
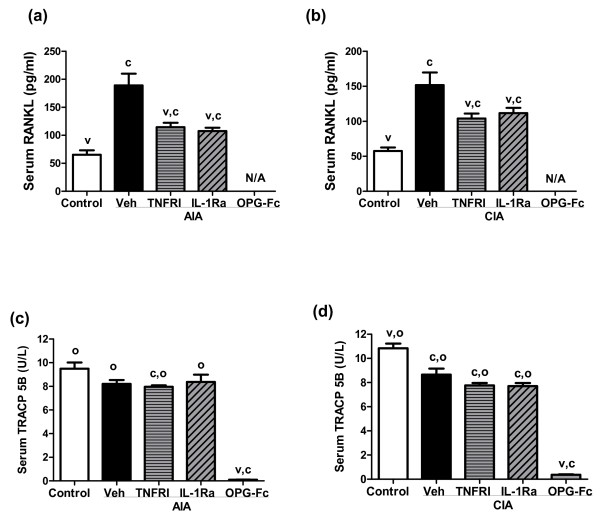
Effect of anti-TNFα, anti-IL-1, or anti-RANKL therapy on bone resorption markers. Effects of PEGylated soluble TNF receptor type I (TNFRI), IL-1 receptor antagonist (IL-1Ra) or osteoprotegerin (OPG)-Fc on serum levels of the bone resorption markers **(a), (b) **receptor activator of NF-κB ligand (RANKL) and **(c), (d) **tartrate-resistant acid phosphatase 5b (TRACP-5B) in (a), (c) adjuvant-induced arthritis (AIA) rats and in (b), (d) collagen-induced arthritis (CIA) rats. Values were determined on day 14 post onset, 10 days after the initiation of treatment. Data represent means ± standard error of the means, n = 8/group. ^c^Significantly different from control (nonarthritic) rats, *P *< 0.05. ^v^Significantly different from vehicle (Veh)-treated arthritic rats, *P *< 0.05. ^o^Significantly different from OPG-treated arthritic rats, *P *< 0.05.

Serum RANKL was significantly increased in vehicle-treated AIA and CIA rats (2.9-fold and 2.6-fold, respectively; *P *< 0.05 versus healthy controls). In AIA and CIA rats, anti-TNFα or anti-IL-1 therapy partially normalized serum RANKL, to levels that were significantly lower than in vehicle-treated arthritic controls but were still significantly higher than in healthy (nonarthritic) controls (Figure 4a, b). AIA or CIA rats treated with human OPG-Fc had 177 ± 23.8 μg/ml circulating OPG-Fc at the end of the study. The interference of pharmacological amounts of OPG-Fc with the capture and/or detection antibodies used in the commercial assays prevented reliable determination of the concentration of endogenous OPG in animals treated with OPG-Fc. Endogenous serum OPG concentrations in AIA and CIA animals (180 ± 67 pg/ml) were not different from those in nonarthritis groups and were not affected by anti-TNFα or anti-IL-1 therapies. Serum TRACP 5B, an osteoclast marker, was not altered in vehicle-treated AIA rats, and was modestly but significantly lower in vehicle-treated CIA rats (*P *< 0.05 versus healthy controls). Anti-TNFα or anti-IL-1 therapy did not significantly alter serum TRACP 5B values in either model, while anti-RANKL therapy reduced serum TRACP 5B by over 90% in both models, thereby confirming that RANKL was significantly inhibited by OPG-Fc (Figure 4c, d).

### Effects of OPG-Fc on serum levels of inflammation markers

**Figure 5:**
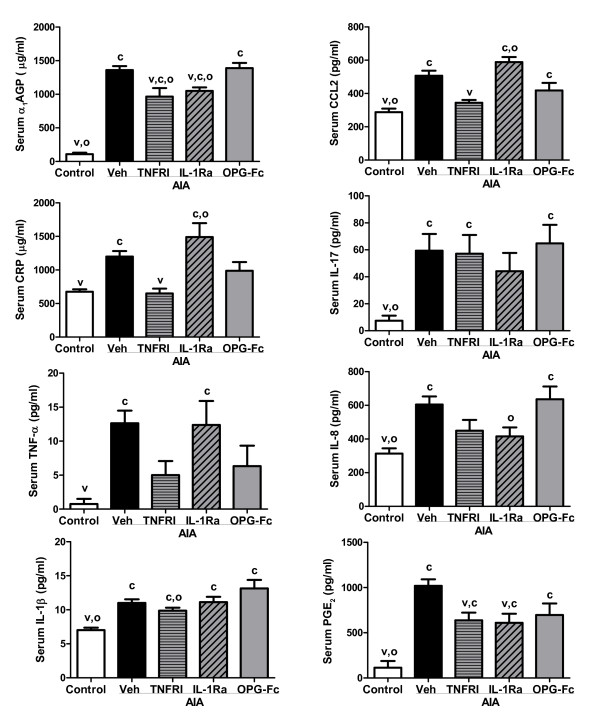
Serum markers and mediators of inflammation in adjuvant-induced arthritis (AIA) rats. Values were determined on day 14 post onset, 10 days after the initiation of treatment. Data represent means ± standard error of the means, n = 8/group. ^c^Significantly different from control (nonarthritic) rats, *P *< 0.05. ^v^Significantly different from vehicle (Veh)-treated arthritic rats, *P *< 0.05. ^o^Significantly different from osteoprotegerin (OPG)-treated arthritic rats, *P *< 0.05. α_1_AGP, acute-phase protein alpha-1-acid glycoprotein; AIA, adjuvant-induced arthritis; CCL2, chemokine (C-C motif) ligand 2; CRP, C-reactive protein; PGE_2_, prostaglandin E_2_.

**Figure 6:**
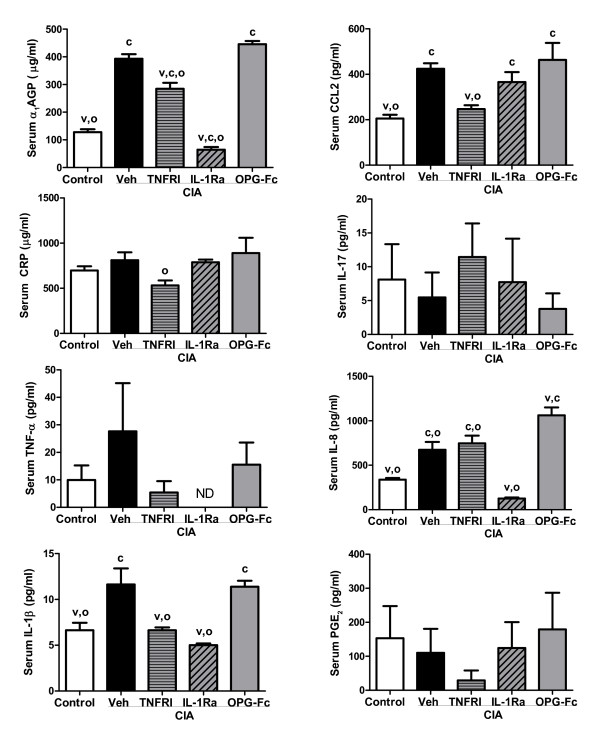
Serum markers and mediators of inflammation in collagen-induced arthritis (CIA) rats. Values were determined on day 14 post onset, 10 days after the initiation of treatment. Data represent means ± standard error of the means, n = 8/group. ^c^Significantly different from control (non-arthritic) rats, *P *< 0.05. ^v^Significantly different from vehicle (Veh)-treated arthritic rats, *P *< 0.05. ^o^Significantly different from osteoprotegerin (OPG)-treated arthritic rats, *P *< 0.05. α_1_AGP, acute-phase protein alpha-1-acid glycoprotein; CCL2, chemokine (C-C motif) ligand 2; CIA, collagen-induced arthritis; CRP, C-reactive protein; PGE_2_, prostaglandin E_2_.

Recent analyses demonstrated that numerous markers and mediators of inflammation are consistently elevated in the AIA and CIA models from day 4 through day 14 after disease onset [38,39]. Figures 5 and 6 provide data on the inflammatory markers that were significantly elevated in vehicle-treated animals from one or both models.

Vehicle-treated AIA rats exhibited significant increases in serum α_1_AGP, CCL2, C-reactive protein, IL-17, TNFα, IL-8, IL-1β, and PGE_2 _(Figure 5). In this model, anti-TNFα significantly reduced α_1_AGP, CCL2, C-reactive protein and PGE_2_, while anti-IL-1 significantly reduced α_1_AGP and PGE_2 _(all *P *< 0.05 versus vehicle-treated controls).

Vehicle-treated CIA rats exhibited significant increases in serum α_1_AGP, CCL2, IL-8, and IL-1β (Figure 6). In this model, anti-TNFα significantly reduced serum α_1_AGP, CCL2 and IL-1β, while anti-IL-1 significantly reduced serum α_1_AGP, IL-8 and IL-1β (all *P *< 0.05 vs. vehicle-treated controls). OPG-Fc treatment had no significant effect on markers or mediators of inflammation in either model, with the sole exception of a 58% increase in serum IL-8 in the CIA rat model. Serum IL-8 levels in OPG-treated AIA rats were similar to levels found in AIA vehicle controls.

### Relationships between serum cytokines, local and systemic bone loss, and joint inflammation

**Figure 7:**
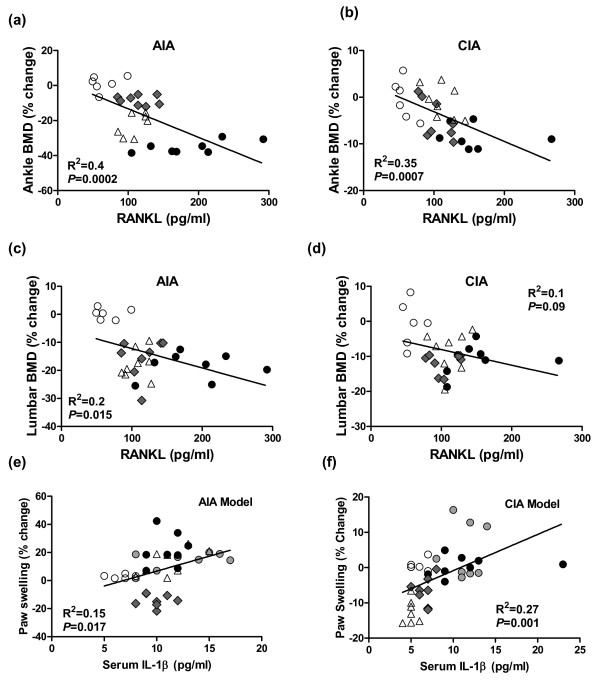
Linear regression analyses of serum cytokines versus local bone loss or local inflammation. **(a) to (d) **Bone loss was quantified as the percentage change in areal bone mineral density (BMD) from the day of onset for clinical arthritis (day 0) to day 14 post onset. **(e) and (f) **Paw swelling was quantified as the percentage change from day 4 post onset (treatment initiation) to day 14 post onset. Serum receptor activator of NF-κB ligand (RANKL) and IL-1β were evaluated on day 14 post onset. Open circles, nonarthritic controls + vehicle; black circles, arthritis + vehicle; open triangles, arthritis + IL-1 receptor antagonist; grey diamonds, arthritis + PEGylated soluble TNF receptor type I; grey circles, arthritis + OPG-Fc. n = 8/group. AIA, adjuvant-induced arthritis; CIA, collagen-induced arthritis.

Linear regression analyses were performed to determine the extent to which serum levels of biochemical markers predicted changes in BMD or paw swelling as a marker of local inflammation. With the exception of the OPG-Fc group, for which RANKL could not be reliably measured, all groups were combined to test the hypothesis that serum RANKL regulates BMD in each model independent of treatment. Consistent with this hypothesis, serum RANKL was significantly and inversely correlated with the percentage change in ankle BMD, with *R*^2 ^values of 0.40 and 0.35 in the AIA and CIA models, respectively (both *P *< 0.001; Figure 7a, b). Serum RANKL was also significantly and inversely correlated with the percentage change in vertebral BMD, with *R*^2 ^values of 0.20 (*P *= 0.015) in the AIA but not in the CIA model (Figure 7c, d). Serum RANKL did not correlate significantly with paw swelling in either model, with *R*^2 ^values of 0.08 and 0.04 in the AIA and CIA models, respectively (regressions not shown). Paw swelling was significantly and positively correlated with serum IL-1β in AIA rats (*R*^2 ^= 0.15, *P *= 0.017) and CIA rats (*R*^2 ^= 0.27, *P *= 0.001) (Figure 7e, f), while serum TNFα correlated with paw swelling in AIA rats (*R*^2 ^= 0.25, *P *= 0.0015) but not in CIA rats (*R*^2 ^= 0.03, *P *= 0.31) (regressions not shown).

## Discussion

The cytokines TNFα, IL-1 and RANKL are commonly elevated, locally and/or systemically, in inflammatory arthritis [31,38,39,42,43]. These molecules are thought to interact in numerous ways that can exacerbate disease. The current studies were conducted to increase understanding of the relative contributions of these proinflammatory and pro-erosive cytokines to the local and systemic components of inflammation and bone loss.

Inhibition of TNFα or IL-1 typically inhibits local inflammation in arthritic animals and patients, while partially preventing local bone loss [10,16,26,44,45]. The differential response of RA patients to anti-TNFα or anti-IL-1 therapies demonstrates that the disease variants in certain RA patient populations are driven preferentially by either TNFα or IL-1. This premise is confirmed by our demonstration in the present study that anti-TNFα is more effective at reducing clinical indices of AIA, while anti-IL-1 is more efficacious against CIA. This outcome is consistent with prior AIA and CIA studies in rats showing that these two models exhibit differential sensitivity to various cytokine blockers [38,39].

Two conundrums in interpreting rodent arthritis studies are the incongruity between anti-inflammatory efficacy as predicted by clinical (that is, paw swelling) versus structural (that is, histopathology) measurements for any given model, and the divergence of anti-arthritic efficacy among different models in the same species. Joint inflammation in rat arthritis models chiefly presents as soft tissue edema superimposed on leukocyte infiltration. Anti-cytokine agents readily alter leukocyte function to thwart the additional release of proinflammatory molecules that increase vascular permeability, but these agents are noncytotoxic and do not completely remove existing inflammatory cell infiltrates.

With respect to the first incongruity, therefore, the divergent clinical and structural responses in a given rodent arthritis model result from the action of cytokine inhibitors to largely alleviate paw swelling while only partially reducing leukocyte influx, even if anti-inflammatory therapy is initiated the day of disease onset [10]. Such residual disease is even more likely if treatment is not initiated until disease has become established, as was done in the present study by delaying treatment until 4 days after disease onset. The second incongruity - the divergence of anti-arthritic efficacy between AIA and CIA - reflects the twin facts that CIA is a milder disease than AIA (Figures 1 and 2, and Table 1) and that progression of the two models is mediated by distinct immunopathogenic mechanisms [41]. With respect to the dominant pro-arthritic cytokines, AIA is driven mainly by TNFα [39] while CIA is impelled principally by IL-1 [38]. Our current data confirm this model-specific dependence on divergent cytokine profiles, as administration of either anti-IL-1 or anti-TNFα in CIA significantly decreased local indices of arthritis (paw swelling and leukocyte infiltration), while anti-IL-1 had no effect on local inflammation and anti-TNFα significantly reduced only paw swelling in AIA (Figures 1 and 2, and Table 1).

Such divergence between clinical versus structural predictors of anti-inflammatory efficacy has also been reported following administration of TNFα blockers to mice with established CIA [46] or following overexpression of human TNFα in transgenic mice with chronic polyarthritis [45]. A probable explanation for this divergence is that TNFα is primarily responsible for tissue swelling [47], presumably in part due to its role in augmented vessel permeability by upregulating synoviocyte and monocyte production of vascular endothelial growth factor [48], while IL-1 is more consequential in regulating leukocyte infiltration and eventual joint destruction [47]. Human clinical experience suggests that the outcomes noted in these rat arthritis models are also typical in human patients in which RA continues to smolder despite the clinical appearance of remission.

RANKL is a pro-resorptive cytokine that is consistently overexpressed in arthritic joints of animals with inflammatory arthritis [31,49]. RANKL can be produced by T cells and can promote the survival of dendritic cells *in vitro*, suggesting a potential immunomodulatory role [50,51]. Anti-RANKL therapy, however, consistently fails to influence inflammatory parameters within arthritic joints of experimental arthritis models, despite its ability to prevent arthritis-related bone loss in the face of ongoing inflammation [21,22,24,26,45,52,53]. Similar findings are evident in RA patients. Despite high expression of RANKL in human arthritic synovium [20], RANKL inhibition via denosumab had no significant effect on measured parameters of inflammation in RA patients [54]. These data suggest that the predominant and perhaps exclusive role of RANKL in arthritis is to promote local and systemic bone loss. There are minimal data, however, on the effects of RANKL inhibitors on *systemic *parameters of inflammation in disease models. In CIA rodents, RANKL inhibition had no significant effects on serum levels of anti-type II collagen [53,55] or on paw swelling [16], but cytokine profiles were not reported.

In two well-characterized rat arthritis models (CIA and AIA), significant increases in circulating levels of numerous inflammatory markers have been described - some of which are overlapping, while others are unique to only one model [38,39]. The clinical stage (from day +4 post onset forward) of both CIA and AIA is characterized by systemic upregulation of acute-phase proteins, IL-1β, IL-8, CCL2 and RANKL, whereas an increase of TNFα, IL-17 and PGE_2 _is elevated exclusively in clinical AIA. We exploited these divergent marker profiles to directly compare for the first time the effects of selective inhibition of RANKL, TNFα or IL-1 on systemic (circulating) markers of inflammation. Both anti-inflammatory therapies (PEGylated soluble TNF receptor type I and IL-1 receptor antagonist) significantly inhibited concentration of acute-phase proteins in serum of AIA and CIA rats. Anti-TNFα therapy was shown to significantly reduce serum PGE_2 _and CCL2 in AIA rats, and to significantly reduce IL-1β and CCL2 in CIA rats. Anti-IL-1 therapy significantly reduced serum PGE_2 _in AIA rats, while significantly reducing IL-8 and IL-1β in CIA rats. Neither of these anti-inflammatory therapies reduced IL-17, one of the major contributors of initiation and progression of arthritis in animal models [39,56].

Recent studies have shown that adenoviral overexpression of IL-17 did not induce joint inflammation in TNFα-deficient mice but did incite the inflammation in IL-1-deficient mice and wild-type controls [57]. This strong IL-17 dependency on TNFα was lost, however, when IL-17 was overexpressed in combination with arthritic stimuli, such as KxB/N serum or streptococcal cell-wall derivatives [57]. Additional IL-1 blockade demonstrated that such loss of TNFα dependency under arthritic conditions was not the result of synergic effects of IL-1 with IL-17 [57]. Hence, despite the strong dependency of IL-17 on TNFα in a naive joint, IL-17 acts both synergistically with and independent of TNF and IL-1 under arthritis conditions [57,58]. Our current observation that anti-TNFα and anti-IL-1 therapies had no effect on increased circulating IL-17 levels in rat AIA supports the hypothesis that IL-17 acts upstream of IL-1 and TNF in experimental arthritis [59,60].

In contrast to anti-TNFα or anti-IL-1 therapies, anti-RANKL therapy with OPG-Fc did not produce any evidence of immunomodulation/immunosuppression at either the local or the systemic levels. The only observed effect of anti-RANKL therapy on the immune system was an increase in IL-8 that was evident in the CIA model. This response might be specific to this disease model, as RANKL inhibition did not influence serum IL-8 in transgenic adult rats (or mice) that continuously overexpressed high systemic levels of OPG [61], nor in AIA rats from this study. IL-8 is produced by monocytes and osteoclasts in arthritis [62], and its major role is thought to be as a chemoattractant for monocytes and granulocytes to inflamed sites [63]. There is also evidence that IL-8 can stimulate the formation and activity of cultured osteoclasts [64]. On the other hand, RANKL - a major maturation factor for osteoclast precursors - is also chemotactic for the recruitment of circulating monocytes at bone remodeling sites [65]. Prior work has also shown that RANKL is chemotactic for human monocytes [65].

Since OPG-Fc therapy inhibits osteoclasts [16] but not local and systemic inflammation in CIA rats, we hypothesized that the 1.6-fold increase in serum IL-8 may be due to the accumulation of osteoclast precursors and/or monocytes in the bloodstream secondary to the inhibition of their osteoclastic differentiation. This hypothesis is consistent with the observation that OPG-Fc treatment in the CIA model (but not in the AIA model) was associated with a modest but significant increase in circulating monocytes (from 7.5% in CIA-Veh to 11% in CIA-OPG-Fc, *P *< 0.05). Our findings also suggested that, in apparent contrast to data from cell culture studies [64], the ability of IL-8 to promote osteoclast formation or activity *in vivo *does require RANKL.

RANKL inhibition by OPG-Fc markedly reduced bone resorption in both models. OPG prevented bone loss locally in inflamed joints and also in far-distant lumbar vertebrae (Figure 3), while causing profound reductions in serum TRACP 5B in both models (Figure 4). Interestingly, anti-TNFα and anti-IL-1 each afforded partial prevention of bone loss in ankle joints in both models, but not in lumbar vertebrae of either model (Figure 3). This apparent difference could be related to the presence of substantial increased inflammatory cell infiltrates in hind paws but not in vertebrae, as previously described in AIA rats [66]. In paws, the direct inhibition of local inflammatory cells by anti-TNFα or anti-IL-1 could have reduced their ability to produce RANKL in response to proinflammatory cytokines, several of which remained significantly elevated despite those therapies. The absence of inflammatory cell infiltrates as therapeutic targets in lumbar vertebrae, coupled with the persistent elevation of serum RANKL in rats treated with anti-IL-1 or anti-TNFα, might explain the lack of significant vertebral BMD preservation with those therapies.

The partial normalization of serum RANKL in rats treated with anti-TNFα or anti-IL-1 is consistent with previous evidence that RANKL is a downstream mediator of bone resorption subject to regulation by TNFα or IL-1 [30,67,68]. Cell culture studies have suggested that TNFα and IL-1 can promote osteoclast formation or activity independently of RANKL [69,70]. Neither IL-1 nor TNFα, however, were capable of stimulating bone resorption in RANK knockout mice [71,72], or in mice treated with OPG [73]. Consistent with those findings, OPG-Fc treatment was able to fully prevent bone loss and markedly reduce TRACP 5B in AIA and CIA rats (Figure 4), even though their serum TNFα and IL-1 levels remained significantly elevated (Figure 5). It therefore remains to be demonstrated that TNFα or IL-1, alone or in combination, can stimulate bone resorption *in vivo *in a manner that is truly independent of RANKL.

The BMD loss associated with AIA was greater in magnitude (-35%) than that associated with CIA (-8.5%). AIA rats also exhibited significant increases in PGE_2 _and IL-17, neither of which was significantly elevated in CIA rats. PGE_2 _has been shown to increase RANKL mRNA in bone marrow cells, and PGE_2 _inhibition via indomethacin reduces RANKL mRNA [74]. Similarly, IL-17 has been shown to stimulate RANKL mRNA expression in synovial fibroblasts, while IL-17 inhibition reduces RANKL expression [29,75]. The disease-specific increases in PGE_2 _and/or IL-17 observed in AIA rats thus might have contributed to the excessive bone loss in this arthritis model relative to CIA. Alternatively, this excessive bone loss in AIA could be related to the significant increase in serum TNFα, which was not observed in CIA rats.

The present study has several limitations. Circulating levels of the osteoclast marker TRACP 5B did not respond to arthritis induction in a manner consistent with the increases in osteoclasts that occurred with both models. A detailed time course of serum TRACP 5B, conducted previously with AIA and CIA rats, also revealed no significant disease-related increases in serum TRACP 5B [31]. The same study, however, demonstrated marked increases in TRACP 5B in protein extracts obtained from inflamed joints [31]. These findings suggest that TRACP 5B reflects bone resorption more accurately when measured locally rather than systemically, at least in these two rat models. Another limitation of the current study pertains to the single time point used for some of the data analyses, and the use of single dose levels for each inhibitor. The timing and duration of treatment were chosen to begin at the peak of clinical disease and to proceed through the major period of disease progression, as established in previous studies [31,38,39]. It therefore remains possible that the local and systemic cytokine profiles, and the responses to each specific anti-cytokine intervention, might vary as a function of treatment timing or the therapeutic dose selected.

## Conclusions

In summary, inhibition of TNFα or IL-1 reduced not only local inflammation but also systemic inflammation as evident by decreased levels of several proinflammatory markers and mediators in two rat models of immune-mediated arthritis. Local inflammation was best predicted by serum IL-1β levels in both models, while serum TNFα also modestly predicted the degree of local inflammation in the AIA model. Anti-TNFα or anti-IL-1 also partially prevented pathologic increases in serum RANKL and local bone loss in inflamed ankles in both models without preventing systemic bone loss in non-inflamed vertebrae. In contrast, the direct pharmacologic inhibition of RANKL by OPG-Fc prevented local (joint) and systemic (vertebral) bone loss in both models, without inhibiting any measured local or systemic parameter of inflammation. Serum RANKL levels predicted the extent of local and systemic bone loss in both models, independent of treatment condition, while showing no correlation with local inflammation. Collectively, these observations add to a growing body of evidence indicating that RANKL does not regulate local inflammation in either AIA or CIA in rats, while further supporting the premise that RANKL is a common and important downstream mediator of local and systemic bone loss in these models [16,21,24-26].

## Abbreviations

α_1_AGP: acute-phase protein alpha-1-acid glycoprotein; AIA: adjuvant-induced arthritis; BMD: bone mineral density; BSA: bovine serum albumin; CCL2: chemokine (C-C motif) ligand 2; CIA: collagen-induced arthritis; ELISA: enzyme-linked immunosorbent assay; Fc: constant domain of immunoglobulin; H & E: hematoxylin and eosin; IL: interleukin; NF: nuclear factor; OPG: osteoprotegerin; PBS: phosphate-buffered saline; PEG = polyethylene glycol; PGE_2_: prostaglandin E_2_; RA: rheumatoid arthritis; RANKL: receptor activator of NF-κB ligand; TNF: tumor necrosis factor; TRACP 5B: tartrate-resistant acid phosphatase 5b; Veh: vehicle.

## Competing interests

MS, DeD, SV, DiD, EP, GV, DZ, and PK are full-time employees of Amgen Inc. and own stock and/or stock options in Amgen Inc. SM, BB, and UF are former employees of Amgen Inc. and own stock and/or stock options in Amgen Inc. GS is an Amgen Inc. collaborator and has no competing interests.

## Authors' contributions

MS made substantial contributions to the conception and design of the study, the analysis and interpretation of the data, and the drafting and critical review of the manuscript. GS made substantial contributions to the conception and design of the study, the analysis and interpretation of the data, and the critical review of the manuscript. DeD made substantial contributions to the acquisition, analysis, and interpretation of the biomarkers data. SV made substantial contributions to the analysis and interpretation of the pathology data and the critical review of the manuscript. SM participated in the design, coordination, and analysis of the *in vivo *experiments. DiD, EP, and GV made substantial contributions to the acquisition and analysis of the pathology data. BB and UF made substantial contributions to the conception and design of the study and the critical review of the manuscript. DZ made contributions to the conception and design of the study, the interpretation of data, and the drafting and critical review of the manuscript. PK made substantial contributions to the analysis and interpretation of data, and the drafting and critical review of the manuscript. All authors reviewed and approved the final manuscript.
